# High-throughput characterization and phenotyping of resistance and tolerance to virus infection in sweetpotato

**DOI:** 10.1016/j.virusres.2023.199276

**Published:** 2023-11-25

**Authors:** Jan F. Kreuze, David A. Ramírez, Segundo Fuentes, Hildo Loayza, Johan Ninanya, Javier Rinza, Maria David, Soledad Gamboa, Bert De Boeck, Federico Diaz, Ana Pérez, Luis Silva, Hugo Campos

**Affiliations:** a International Potato Center (CIP), Headquarters, P.O. Box 1558, Lima 15024, Peru; b Programa academico de ingenieria ambiental, Universidad de Huanuco, Jr. Hermilio Valdizan N° 871, Huanuco, Peru

**Keywords:** Ipomoea batatas, LAMP, Machine learning, Remote sensing, SPFMV, SPCSV, SPLCV, Virus detection

## Abstract

Breeders have made important efforts to develop genotypes able to resist virus attacks in sweetpotato, a major crop providing food security and poverty alleviation to smallholder farmers in many regions of Sub-Saharan Africa, Asia and Latin America. However, a lack of accurate objective quantitative methods for this selection target in sweetpotato prevents a consistent and extensive assessment of large breeding populations. In this study, an approach to characterize and classify resistance in sweetpotato was established by assessing total yield loss and virus load after the infection of the three most common viruses (SPFMV, SPCSV, SPLCV). Twelve sweetpotato genotypes with contrasting reactions to virus infection were grown in the field under three different treatments: pre-infected by the three viruses, un-infected and protected from re-infection, and un-infected but exposed to natural infection. Virus loads were assessed using ELISA, (RT-)qPCR, and loop-mediated isothermal amplification (LAMP) methods, and also through multispectral reflectance and canopy temperature collected using an unmanned aerial vehicle. Total yield reduction compared to control and the arithmetic sum of (RT-)qPCR relative expression ratios were used to classify genotypes into four categories: resistant, tolerant, susceptible, and sensitives. Using 14 remote sensing predictors, machine learning algorithms were trained to classify all plots under the said categories. The study found that remotely sensed predictors were effective in discriminating the different virus response categories. The results suggest that using machine learning and remotely sensed data, further complemented by fast and sensitive LAMP assays to confirm results of predicted classifications could be used as a high throughput approach to support virus resistance phenotyping in sweetpotato breeding.

## Highlights

• Sweetpotato is classified in resistance categories by yield loss and virus load.
• Rapid isothermal amplification was accurate to confirm virus load.
• Sweetpotato resistance can be accurately predicted using remotely sensed data.

## 1 Introduction

Virus infections represent one of the most critical production constraints for sweetpotato (*Ipomoea batatas* L.) worldwide (Clark et al., 2012) and particularly in low- and middle-income countries where there is a lack of clean seed and farmers mostly recycle their planting material, leading to accumulation of virus infections. Therefore, increasing virus resistance is a significant focus in sweetpotato breeding worldwide (Kreuze et al., 2021). In farmer fields, sweetpotatoes are exposed to many different viruses that often cause minor damage and symptoms by themselves but have severe impacts when they occur in combinations. (Untiveros et al., 2007; Wanjala et al., 2020). Sweetpotato feathery mottle virus (SPFMV; genus *Potyvirus,* family *Potyviridae*) and sweetpotato chlorotic stunt virus (SPCSV; genus *Crinivirus,* family *Closteroviridae*) are the most common viruses found infecting sweetpotato globally and also the best studied. Together they cause a severe synergistic disease referred to simply as sweet potato virus disease (SPVD; Karyeija et al., 2000a). On the other hand, sweetpotato leaf curl virus (SPLCV; genus *Begomovirus,* family *Geminiviridae*) related begomoviruses have recently been recognized as the third most frequent virus in sweetpotato and is also synergized by SPCSV (Cuellar et al., 2015), but has been much less studied in this respect. Among sweetpotato infecting viruses, SPCSV is particularly relevant as it is able to cause synergistic diseases with all other sweetpotato viruses tested. This is the result of the action of a type 1 Ribonuclease III enzyme (RNase3) encoded by SPCSV (Cuellar et al., 2009), leading to suppression of RNA silencing mediated viral defense in sweetpotatoes, through cleavage of small interfering RNA molecules and interaction with the host encoded suppression of gene silencing 3 (SGS3) protein (Weinheimer et al. 2014, 2016). This knowledge has led to development of strategies to inhibit RNase3 using small molecule inhibitors or through CRISPR-Cas13 targeting, thereby reducing disease (Wang et al., 2021; Yu et al., 2022). However, natural occurring resistance to SPCSV and other sweetpotato viruses is currently the only practical approach to control the disease. Whereas very high levels of resistance have been identified to SPFMV (Karyeija et al., 2000b), these are lost completely upon co-infection with SPCSV, and strong sources of resistance to SPCSV have remained elusive. On the other hand, cheap and rapid diagnostics like ELISA often need to be more sensitive in sweetpotato to identify uninfected plants reliably. Thus, breeders select for resistance by evaluating symptoms and yield in the field over several seasons compared to check/control varieties using a standardized but subjective scale (Grüneberg et al., 2019). In addition, virus resistance in sweetpotato is polygenic and quantitative (Mwanga et al., 2002a), resulting in relatively few genotypes with acceptable resistance levels in any given parental combination. The ability to precisely quantify virus titers and disease phenotyping in sweetpotato genotypes would likely accelerate the genetic gains to be achieved through virus resistance in sweetpotato breeding programs.

Since several mechanisms can lead to a resistant phenotype, to develop a more quantitative method of resistance evaluation a more objective set of definitions for the type of plant response to virus infection than breeders currently use is required. Cooper & Jones (1983) proposed a set of terms for resistance, susceptibility, tolerance, and sensitivity that are broadly suitable. Susceptible genotypes allow the virus to accumulate to high concentrations resulting in disease (symptoms and yield loss), whereas sensitive genotypes suffer disease despite low virus concentrations. On the other hand, resistant genotypes limit pathogen multiplication, thereby reducing the damage and disease (with no or mild symptoms and limited or no yield reduction), whereas tolerant genotypes can yield well, even if viruses infect them and accumulate to high concentrations. Resistance to virus inoculation by insect vectors is also crucial under natural conditions. Current virus selection schemes are unable to distinguish between these types of resistance. Approaches that consider tolerance and resistance for selection require methodologies that allow the assessment of an extensive panel of genotypes in a relatively short time and low cost.

In this study, we explore several approaches to improve rapid sweetpotato virus resistance phenotyping for the three most common sweetpotato viruses mentioned SPFMV, SPCSV and SPLCV under both artificial and natural infection pressures under field conditions with 12 diverse genotypes with contrasting reactions to virus infection. Specifically, we evaluate loop-mediated isothermal amplification (LAMP) as a rapid and cheap alternative to determine virus load and remotely sensed data from an unmanned aerial vehicle to enable rapid quantitative determination of viral symptoms. The specific objectives of this study were: i) to assess LAMP accuracy in virus load determination compared with quantitative PCR (qPCR), and ii) to associate temporal remotely sensed data to sensitive, susceptible, tolerant, and resistant genotypes to combined infection by SPFMV, SPCSV, and SPLCV utilizing machine learning algorithms.

## 2 Materials and methods

### 2.1 Study area, plant material, and grafted virus infection

The study was carried out at San Ramon – International Potato Center (CIP) experimental station in the Peruvian central Amazon in Chanchamayo Province, northern Junin region (11° 7’ 39’’ S, 75° 21’ 23’’ W, 850 m.a.s.l.) from 10 September 2021 to 15 February 2022. This site is characterized by a rainy, warm, and humid climate throughout the year, with a maximum temperature, minimum temperature, and annual precipitation ranging between 29 – 33 °C, 19 – 23 °C, and 2000 – 3500 mm, respectively (SENAMHI, 2022). The soil has a sandy-loam texture (64, 20, and 16 % of sand, silt, and clay, respectively) with a content of phosphorus, potassium, and organic matter of 36.4 ± 2.80 ppm, 100.2 ± 2.20 ppm, and 1.42 ± 0.12 %, respectively (Soil, Plant, Water, and Fertilizer Analysis Laboratory of National Agrarian University–La Molina, Lima, Peru). During the growing season, the average relative humidity, average air temperature, and accumulated rainfall were 82.1 ± 0.49 %, 23.8 ± 0.13 °C, and 926.6 mm, respectively (Rinza et al., 2021). The twelve studied sweetpotato genotypes (Table S1) included globally important released varieties (Beauregard, Benjamin, Arne, Dagga), regional cultivars (Ejumula, Moch, SPK004/1-Kakamega, Tanzania, New Kawogo) and advanced clones (VZ08.294, VZ08.328, VZ08.391) selected from a population segregating for virus resistance. These genotypes represented a panel with contrasting susceptibility levels to sweetpotato virus disease (representing the combined impact of a combination of viruses present in the environment), as assessed by breeders. Twenty clean in-vitro plantlets of each genotype (except for the “VZ” clones and ‘Dagga’ maintained in greenhouses as healthy material) from CIP's gene bank were propagated in Jiffy grow pellets for 20 days and irrigated three times a week. Then, plants were transferred to 2.5 L pots with a peat-based substrate (Sogemix SM-2 Premier tech, Canada) for 45 days in a screen house, irrigated at saturation three times per week, and fertilized with 27, 15, and 12 % of N-P-K, respectively. Plant propagation included pest control for insects and snails every two weeks, pruning according to growth, and foliar fertilization every two weeks. After four months of propagation, 500 plants per genotype were obtained. In parallel, inoculum was prepared by simultaneously wedge grafting a susceptible sweetpotato plant (cv. Beauregard) with scions of *Ipomoea setosa* infected with SPFMV isolate Piu (Genbank EU021072), SPCSV isolate m2-47 (Genbank HQ291259, HQ291260), and SPLCV isolate sp54 (Genbank OR344912), from CIP's virus collection and originally isolated from field infected plants in Peru. After successful infection the triple infected plant was multiplied through stem cuttings and served as primary inoculum for graft infection of the different genotypes as well as spreader rows for natural infection in the field trial.

A group of 1980 plantlets (165 per genotype) was transported to separate screen house for virus inoculation by wedge grafting at the position of an axillary bud, with a single node, including leaf, of the previously prepared triple infected ‘Beauregard’. Infected plants were tested randomly by PCR to confirm presence of the viruses as well as confirmed by symptoms. All plantlets (inoculated and not inoculated) were grown from fresh cuttings for 30 days until transplanting into the field.

### 2.2 Field trial layout and management

Three treatments were tested in 740 m^2^ (63.9 m x 11.9 m) blocks: i) Control: vines were transplanted 205 m away from other treatments where the block was further protected with a maize border, and plants were frequently sprayed to prevent insect vectors (see below); ii) Natural Infected (NI): vines were transplanted in an open block surrounded by infected plants (spreader rows) and close to a graft-infected block (as virus source) with no protective border, and plants were not fumigated, iii) Grafted-Infected (GI): transplanted vines belong to the inoculated plantlets with no protective border, and plants were not fumigated (see 2.1) to allow vector insects move between GI and NI for virus transmission. Thirty-six plots (4.5 m x 3.3 m) were established for each treatment block. The 12 genotypes were assigned to the plots using a randomly generated resolvable row-column design with three replications. The three (complete) replication groups comprised 3 rows and 4 columns and were contiguous along the block. Thus, 3 rows and 12 columns were present in each block. Additionally, the designs were latinized by the rows, meaning each genotype occurs once in each row. Plots in each block were separated 1 m from each other. Fifty-five vines per genotype were transplanted per plot at 0.3 m and 0.9 m between plants and rows, respectively (at a density of 3.7 plants m^−2^). Fertilization was carried out at transplanting time at a dose of 67.3 kg NPK (N: P_2_O_5_: K_2_O) ha^−1^. Additionally, at the time of hilling (57 days after transplanting-DAT), a dose of 193.4 kg ha^−1^ of N was applied. For the control block, chemical applications for insect control were made twice (35 and 54 DAT) by spraying Lorsban 4E (Dow AgroSciences, Italy) and Confidor 350 SC (Bayer AG, Germany) with 2 mL L^−1^ and 1.3 mL L^−1^ of doses, respectively; and 1 time with Confidor 350 SC at 67 DAT. Sprinkler irrigations were applied at 0, 4, 10, 21, 42, and 57 DAT with 25, 25, 25, 24.1, 31.7, and 45.5 mm, respectively.

### 2.3 Virus load determination

After field transplanting, leaf sampling and virus load determinations were done 48 and 83 DAT. Ten leaves (from the middle part of each plant) from ten individual plants were collected from each plot following an “X” sampling pattern, taking five leaves from each diagonal transect at equidistant intervals. The leaves from each transect were sampled by laying them over each other and taking a circular punch (using a 1.5 mL-Eppendorf tube) through all leaves to create two bulk samples per plot. This resulted in 72 bulked samples per treatment (12 genotypes x 2 bulked samples x 3 replications). Punches were taken from opposite sides of the main leaf vein for nucleic acid extraction or ELISA tests at 48 DAT. A third punch was taken over the central vein at 83 DAT to have an additional sample for alkaline polyethylene glycol extraction (see below).

For the first determination, miRNA kits (OMEGA bio-tek, USA) were used to purify nucleic acid for LAMP and qPCR assays. The second sampling used APEG buffer (2 mL for each bulked sample) as a quick nucleic acid extraction method (Wanjala et al., 2021) for LAMP, and miRNA kits for purifying nucleic acid for qPCR assays. A separate experiment was performed to compare LAMP performance after alkaline polyethylene glycol and kit extraction from an identical sample. Leaves were collected from 13 randomly selected healthy and diseased-looking plants from a field at CIP-La Molina experimental station (12° 4′ S, 76° 56′ W, 244 m.a.s.l.) as well as healthy and infected controls from CIPs virus collection. Collected leaves were desiccated by sealing them in Ziplock bags with silica gel for one week and then ground them into a homogenous powder using a Bullet Blender Storm Pro (Next Advance, USA) for each separate plant. Twenty mg of homogenized leaf powder was then processed using alkaline polyethylene glycol or miRNA kits and LAMP reactions were run simultaneously in parallel for all samples and the three viruses.

For ELISA tests, antibodies for SPFMV-RC were processed at CIP to obtain immunoglobulin G (IgG) and prepare conjugate (alkaline phosphatase linked to IgG). SPCSV antibodies (Pab and Mab Mix-1) were obtained commercially (DSMZ, Germany). ELISA was performed according to Clark & Adams's (1977) method, and absorbance values were recorded at 405 nm using an Elx800 ELISA reader (BioTek, USA) twice after 30 min and 1 h.

Primers and probes used in the LAMP and (RT-)qPCR assays are listed in Table S2. An additional primer–probe set, based on plant cytochrome oxidase (COX), was used as a positive internal control to assess the quality of the nucleic acid extracts and to normalize virus amplification results in case of (RT-)qPCR. LAMP and (RT)-qPCR assays were run in a Stratagene Mx3005P real-time instrument (Stratagene, USA). For (RT-)qPCR and LAMP, the TaqMan Universal PCR Master Mix (Applied Biosystems, UK) and ISO-001 LAMP Isothermal Mastermix (Optigene, UK) were used, respectively. The cycling reaction for RT-qPCR was 42 °C for 30 min (1 cycle for reverse transcription), 95 °C for 10 min (1 cycle for inactivating the reverse transcriptase and activating the polymerase enzyme), and 95 °C for 15 s and 60 °C for 60 s (40 cycles, for amplification). LAMP reaction was performed at 65 °C for 40 min (1 cycle per min), followed by a melting step from 75 to 95 °C. Both (RT-)qPCR and LAMP reactions were set up in a 25 µL-volume (Supplementary Table S3). The C(t) values automatically determined by the MxPro QPCR software (Stratagene, USA) were used for downstream analysis except that where no C(t) was recorded (i.e., a negative result) the C(t) was set at the maximum cycle number of 40.

### 2.4 Symptom scoring, remote sensing, natural abundance of C^13^, and total yield assessments

Photos were taken from each plot, and representative plants and leaves of each plot with an iPhone 13 or Lumix digital camera (Panasonic, Model DMC-FZ60, Japan) and symptoms were scored according to the scale from 1 to 9 used by breeders to assess virus severity (Grüneberg et al., 2019): 1 = No virus symptoms; 2 = Unclear virus symptoms; 3 = Clear virus symptoms for < 5 % of plant per plot; 4 = Clear virus symptoms for 6–15 % of plants per plot; 5 = Clear virus symptoms for 16–33 % of plants per plot; 6 = Clear virus symptoms for 34–66 % of plants per plot (i.e. > 1/3 and <2/3); 7 = Clear virus symptoms for 67–99 % of plants per plot (2/3 to almost all); 8 = Clear virus symptoms for all plants per plot (not stunted); and 9 = Severe virus symptoms for all plants per plot (stunted). A thermal and multispectral camera (Altum model, MicaSense Inc., Seattle, WA, USA) was assembled and attached to a quadcopter (Inspire 2 model, DJI, Shenzhen, China) to take 16-bit multispectral images with five spectral bands (blue: 475 ± 20 nm, green: 560 ± 20 nm, red: 668 ± 10 nm, near-infrared: 840 ± 40 nm, and red-edge:717 ± 10 nm) at 3.2 megapixels resolution (2064 × 1544 pixels) and long-wave infrared thermal-based at 160 × 120 pixels resolution. The images were acquired at 40 m above ground level using a flight plan that enabled 75 % overlap between the images in auto-shoot mode at a speed of 3 m s^−1^. The reflectance of each multispectral band was computed using a white reference panel (MicaSense Inc., Seattle, WA, USA) while the manufacturer radiometrically calibrated the thermal images. Two flights were conducted at 79 and 95 DAT. The ortho-mosaics were obtained using commercial photogrammetry software (Pix4D SA, Switzerland). These were segmented using an open-source Geographic Information System software (QGIS Development Team, 2020) to get only vegetation data per plot. The mean values per plot of green, red, red-edge, and near-infrared reflected bands and the minimum, maximum and mean value of apparent canopy temperature were used as inputs to classify the 12 sweetpotato genotypes using machine learning algorithms (see Section 2.5). In addition, vegetation indexes were computed as normalized difference red edge index – NDRE (Barnes et al., 2000) and green normalized difference vegetation index – NDVIg (Gitelson et al., 1996). At 80 DAT, ten randomly selected young sun-exposed leaves were collected per plot. Sampled leaves were dried at 60 °C for 48 h, ground in a ball mill (BMIX-100 model, MRC Holon, Israel), and packed in tin capsules. Natural ^13^C/^12^C isotope abundance was assessed using an elemental analyzer (PDZ Europa ANCA-GSL, Sercon Ltd, UK) interfaced with an isotope ratio mass spectrometer (PDZ Europa 20-20, Sercon Ltd, UK) at UC Davis Stable Isotope Facilities. Leaf ^13^C discrimination (Δ) calculation was done according to Ramírez et al. (2015). This trait reflects an integrated photosynthetic performance value for sweetpotato leaves (Ramírez et al. 2017).

Harvest occurred at 159 DAT; all the plants in the 3 inner rows (33 plants) were collected from each plot for yield data collection. The foliage and storage roots were separated by plant and weighed on an electronic platform balance (LP7510, Locosc, China). The total yield was calculated by dividing their total weight (foliage and storage roots in kg) by 8.91 m^2^ which was the area of the plot without borders and multiplied by 10 to convert to t ha^−1^. The percental variation of total yield to control for a genotype (Δ%) was estimated as follows:

(1) $$\Delta \%=\;\frac{TY_{x}-TY_{c}}{TY_{c}}*100$$

where TY_C_ is the average total yield of the assessed genotype in the control block, TY_X_ is the total yield for this genotype in a plot belonging to treatments GI or NI.

### 2.5 Data analysis

ELISA absorbance values were subtracted from those of the negative control before further analysis. To compare (RT-)qPCR and (RT-)LAMP results, inverse threshold cycles (C(t)) were used as a proxy for viral loads, where the C(t) was subtracted from the maximum number of cycles (measured each minute in the case of LAMP) set at 40. This was done because the amplification kinetics of LAMP is not yet fully understood (Dangerfield et al., 2023) in contrast to the cyclic amplification in qPCR and thus methods such as (ΔC(t)) (Livak & Schmittgen, 2001), that apply to qPCR cannot be used for LAMP. Inverse C(t) values were also compared to ELISA results. To quantify the relationship between the qPCR C(t) measurements and the LAMP C(t) measurements, a Deming regression was conducted to regress the LAMP measurement results on the qPCR measurements, and this for all 3 viruses and both evaluations separately (because of the difference in sampling method for LAMP across evaluations). The same was done to compare ELISA measurements to the qPCR reference method. The “mcr” R package (v1.3.2; Potapov et al., 2023) was used to fit these regression models. Estimated intercept and slope indicated systematic and proportional differences between both measurement methods. Using a bootstrap-based method, 95 % confidence intervals of both regression parameters were obtained, and a 95 % confidence area of the regression line, quantifying the (un)certainty of the estimated regression line, and the ability of a linear calibration curve representing the association between both measurement methods. qPCR was considered the gold standard to determine the relative viral load in samples, and the ΔC(t) method was applied (Bio-Rad, 2006). The ΔC(t) method provides relative expression ratio (RER) values, generating results identical to those from Livak & Schmittgen's (2001) method, by calculating the difference between the reference (C(t)_COX_) and the target (C(t)_sample_) C(t) values for each sample as follow:

(2) $$Relative\;expresion\;\left(RE\right)=2^{\left[C{\left(t\right)}_{cox}-C{\left(t\right)}_{sample}\right]}$$

(3) $$RE\;ratio\;\left(RER\right)=\frac{RE}{RE_{calibrator}}$$

**Fig. 1:**
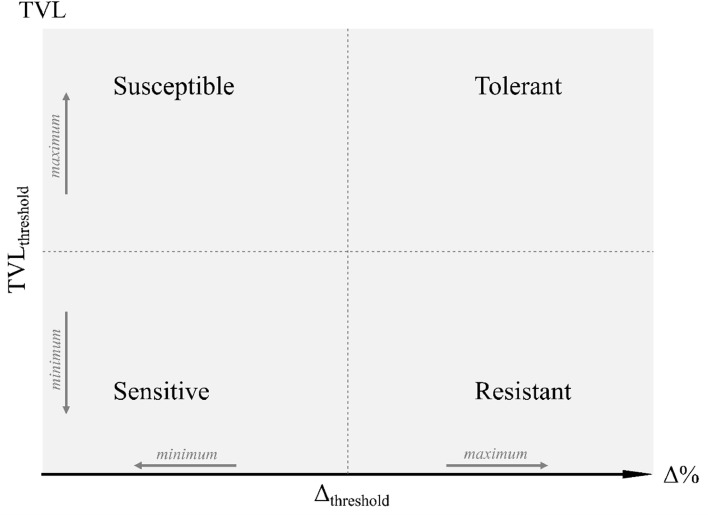
Schematic representation used for sweetpotato genotypes classification based on total viral load for SPFMV, SPCSV, and SPLCV (TVL), and percentage of total yield reduction to control (Δ%). Four categories were determined: Sensitive, Susceptible, Resistant, and Tolerant. The midpoint of the range of TVL values in the grafted-infected treatment as TVL_threshold_ and -20 of Δ% as Δ_threshold_ were used to define the classification axis for establishing the quadrants.

As a calibrator, a sample with C(t) value 40 and the lowest RE was selected. A classification based on the total viral load (TVL, calculated as the arithmetic sum of qPCR RER values for SPFMV, SPCSV, and SPLCV) and total yield reduction (Δ%) relationship was established for all plots belonging to NI and GI blocks according to the terms proposed by Cooper & Jones (1983). Total yield was used because it reflects the overall impact of virus infection on photosynthetic production capacity of the plant, rather than possible effects on within plant partitioning of photo-assimilates (to roots or foliage) in the plant which may depend on the breeding targets and which can vary according to the product profile, including high vine production (for fodder), high root production (for human consumption) or both (dual purpose sweetpotatoes). Four quadrants of the TVL *vs.* Δ% scatter plot were generated considering the midpoint of the range of TVL values as the threshold for the y-axis (TVL_threshold_), and the value of -20 % of Δ% as the threshold for the x-axis (Δ_threshold_) (Fig. 1). Four categories were established based on the quadrants as follows: i) Sensitive: located in the lower left quadrant, with reduced biomass accumulation (Δ% < Δ_threshold_) and low virus load (TVL < TVL_threshold_), ii) Susceptible: plots in the upper left quadrant, with reduced biomass accumulation (Δ% < Δ_threshold_) and high virus load (TVL > TVL_threshold_), iii) Resistant: located in the lower right quadrant, with high biomass accumulation (Δ% > Δ_threshold_) and low virus load (TVL < TVL_threshold_), iv) Tolerant: located in the upper right quadrant, with high biomass accumulation (Δ% > Δ_threshold_) and high virus load (TVL > TVL_threshold_). A global classification for the assessed genotypes was performed using GI treatment, assuming that they can express their maximum response capacity to virus infection under this condition. Genotypes were classified into one of the four categories if 2 of the 3 plots fell into the associated quadrant.

To train machine learning algorithms to classify plots based on remotely sensed data, each genotype was assigned into categories Sensitive, Susceptible, Resistant and Tolerant, respectively. Three machine learning methods were tested: random forest, support vector machine, and K-nearest neighbors, coded in Python programming language (Van Rossum & Drake, 2009). For each method, 4 binary classifiers corresponding to the 4 categories (Sensitive, Resistant, Susceptible, and Tolerant) were created. To evaluate the performance of each classifier, an accuracy assessment was computed (Rogers & Girolami, 2016). To compute a global result, the 4 classifiers were converted to a single multiclass classifier using the maximum argument (Argmax) method (Ravichandiran, 2019). A total of 108 viral loads and total yield assessments were performed. Therefore, to apply machine learning algorithms, the previously established global classification was divided randomly only into two groups: 67 % for the training and 33 % for testing. Additionally, before applying machine learning algorithms, “Mutual Information” was used to measure the level of connections between the predictors (features) and the categories (target) on the dataset (Vergara & Estévez, 2014). The features with the most information of the two aerial assessments were 14 in total, i.e., the mean values evaluated over all pixels of each plot on red, green, red-edge and near-infrared multispectral bands, and the minimum, maximum, and mean apparent canopy temperature (CT at 79 and 95 DAT). These were identified through “Mutual Information” score, thus reducing the dimensionality of the input feature vector. Finally, the Univariant Feature Selection method (Jain & Saha, 2022) based on univariant statistical tests was used to select the most significant feature in the dataset.

## 3 Results

All data and metadata, processed and raw, have been deposited in Dataverse (Loayza et al. 2023; Rinza et al., 2021).

### 3.1 Plant responses to treatments

**Fig. 2:**
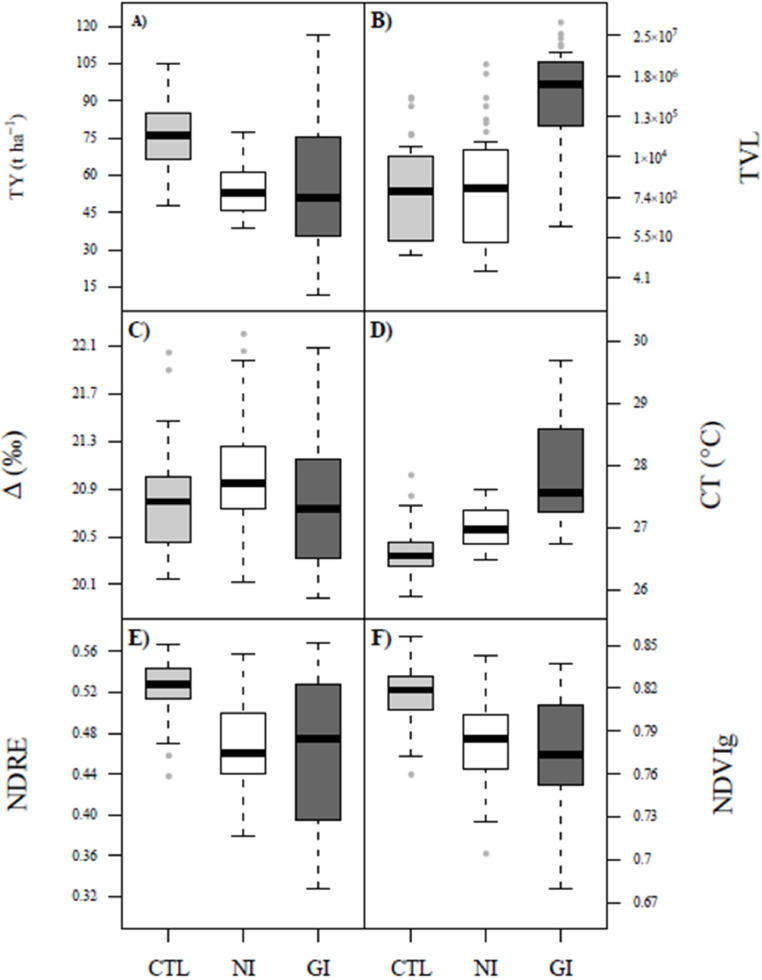
Boxplots of total yield (TY, A), total virus load (TVL, B), leaf ^13^C discrimination (Δ), C), canopy temperature (CT, D), normalized difference red edge index (NDRE, E) and green normalized difference vegetation index (NDVIg, F) for three treatments (CTL – Control, NI – Natural-infected, and GI – Grafted-infected). Remote sensing indicators (CT, NDRE and NDVI_g_) and Δ were acquired/assessed at 79 days after transplanting (DAT). TVL was assessed during 48 and 79 DAT.

**Fig. 3:**
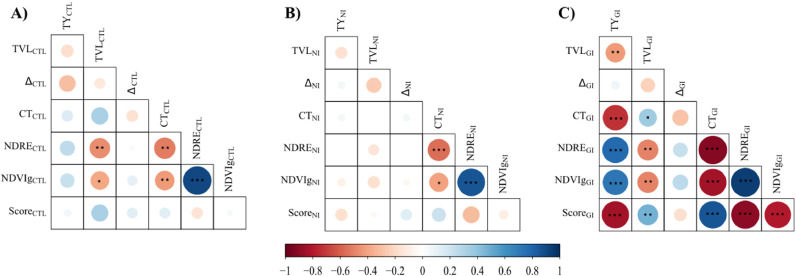
Pearson correlation index values among total yield (TY), total virus load (TVL), leaf ^13^C discrimination (Δ), canopy temperature (CT), normalized difference red edge index (NDRE), green normalized difference vegetation index (NDVIg), and symptom score (Score) for the CTL – control (A), NI – natural-infected (B), and GI – grafted-infected (C) treatments. *, **, and *** means a significant linear relationship between variables at p-value < 0.05, 0.01, and 0.001, respectively. Remote sensing indicators (CT, NDRE and NDVI_g_) and Δ were acquired/assessed at 79 days after transplanting (DAT). TVL was assessed during 48 and 79 DAT.

Total yield, TVL, Δ, canopy temperature, NDRE, and NDVIg values ranged between 27.3–216.3 t ha^−1^, 6.1–5.7 × 10^7^, 19.98–22.20 ‰, 25.9–29.7 °C, 0.33–0.57, and 0.68–0.86, respectively. The control showed higher total yield, NDRE, and NDVIg than NI and GI treatments (Figs. 2 A, E, F). TVL and canopy temperature values presented the following pattern: GI > NI > control (Figs. 2 B, D). Δ in NI was higher than control and GI-infected treatments (Fig. 2C). The main significant correlations were detected under GI treatment (Fig. 3). Thus, symptoms score showed a strong negative and significative (p-value < 0.001) correlation with total yield (r_Pearson_= -0.84), NDRE (r_Pearson_= -0.90), and NDVIg (r_Pearson_= -0.80); and a strong positive and significative (p-value < 0.001) correlation with canopy temperature (r_Pearson_= 0.85) (Fig. 3C). Total yield was strongly positively correlated with NDRE (r_Pearson_= 0.78, p < 0.001) and NDVIg (r_Pearson_= 0.73, p < 0.001), and strongly negatively correlated with canopy temperature (r_Pearson_= -0.72, p < 0.001) (Fig. 3C). Canopy temperature showed strong negative significant (p < 0.001) correlations with NDRE, and NDVIg (r_Pearson_ -0.92 and -0.83, respectively), whereas TVL showed positive and negative correlations (p < 0.01) with symptom score (r_Pearson_ = 0.36) and reflectance indexes (r_Pearson_ = -0.39 to -0.37), respectively (Fig. 3C). In all treatments, NDRE and NDVIg showed high positive correlations (r_Pearson_ 0.86 to 0.94, p < 0.01) (Fig. 3).

### 3.2 Virus loads and comparison between ELISA, LAMP and qPCR methods

**Fig. 4:**
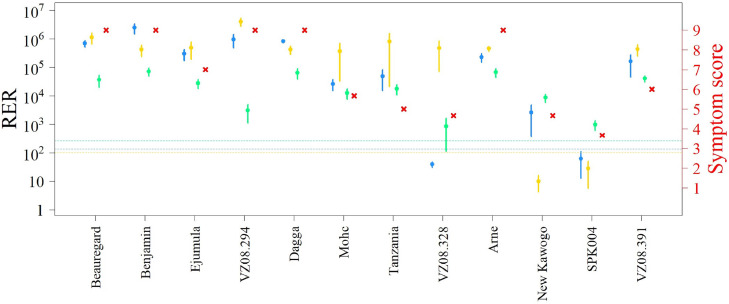
Average (± standard error) of relative expression ratio of viruses (RER, logarithmic scale) and symptom score (red cross) in graft-infected sweetpotato genotypes, assessed at 48 days after transplanting in the Graft Infected treatment. Sweet potato feathery mottle virus (SPFMV, blue), sweet potato chlorotic stunt virus (SPCSV, yellow), and sweet potato leaf curl virus (SPLCV, green). Colored doted lines correspond to C(t) values of 35 for each virus, below which values approach background levels.

**Fig. 5:**
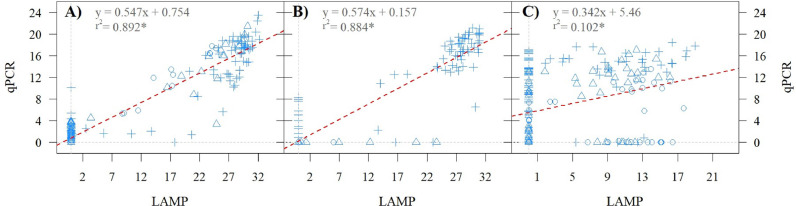
Correlation between inverse C(t) values of LAMP and qPCR methods used for the detection of SPFMV (A), SPCSV (B) and SPLCV (C) in the graft-infected (+), natural infected (Δ), and control (○) treatments at 48 days after transplanting. * p<0.001

RERs showed a large variation spanning several orders of magnitude within and among genotypes (Fig. 4). Typically, relative virus loads followed similar patterns for the three viruses in the different genotypes, i.e. genotypes with high relative virus titers for one also presented high virus titers for the other two viruses, the major exceptions being VZ08.294, VZ08.328 and New Kawogo (Fig. 4). Viral loads (TVL) were positively correlated with symptom severity scores determined by humans (Score) for the NI and GI treatments (Figs. 3 B, C). Results of ELISA tests were correlated to those of the inverse C(t) of LAMP and qPCR reactions for SPFMV and SPCSV. As expected, many more samples were positive for the latter two methods compared to ELISA (Fig. S1). Compared to qPCR, ELISA positive results appeared mostly in samples with inverse C(t) greater than about 5 and 14 for SPFMV and SPCSV, respectively, although there were also some false positive measurements obtained by ELISA for SPFMV (when considering A_405_>0.1 as a confident positive reaction). This corresponds to approximately 30- and 4000-fold difference concentrations that can be detected by qPCR as compared to ELISA for each virus, respectively. Compared to LAMP, positive ELISA reactions generally only appeared for LAMP reactions above inverse C(t) values of 10 in the case of SPFMV, and 25 in the case of SPCSV. When comparing inverse C(t) values of LAMP and qPCR reactions run from identical RNA extractions during the first evaluation (at 48 DAT) a high positive direct correlation could be observed for SPFMV and SPCSV, but not SPLCV (squared Pearson's correlation coefficient r^2^ =0.89, 0.88 and 0.10, respectively; Fig. 5). This correlation was however strongly reduced (r^2^ =0.28, 0.59 and 0.00 for SPFMV, SPCSV and SPLCV, respectively) in the second evaluation (83 DAT) where different leaf punches and extraction methods were used for LAMP and qPCR assays. The fitted Deming regression lines (Fig. S2), confirm that the (linear) association between LAMP and qPCR was strongest for virus detection at the first evaluation (at 48 DAT). We can also observe that the (linear) associations between LAMP and qPCR are much stronger for SPFMV and SPCSV compared to SPLCV detection, as this is reflected by narrower 95 % confidence areas in Fig. S2. When comparing inverse C(t) values resulting from LAMP reactions run on APEG or miRNA kit extracted samples from identical source material (homogenized powdered leaves, see materials and methods), a high correlation of 0.91 and 0.99 was found for SPFMV and SPCSV, respectively (Fig. S3).

### 3.3 Genotype classification under potential virus infection and main remotely sensed predictors

**Fig. 6:**
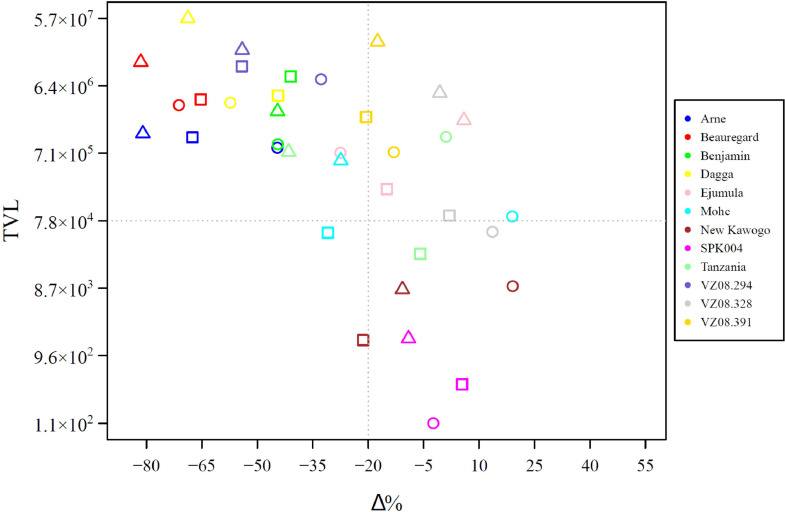
Genotypes classification based on total viral load (TVL) for SPFMV, SPCSV, and SPLCV, and percentage of total yield reduction to control (Δ%) relationship corresponding to the grafted-infected treatment. Triangles, circles, and squares represent repetitions (plots) corresponding to each genotype.

Arne, Beauregard, Benjamin, Dagga, and VZ08.294 were classified in the Susceptible category; Ejumula, VZ08.391, and VZ08.328, were classified in the Tolerant category; and SPK 004 and New Kawogo were categorized in the Resistant category (Fig. 6). It was not possible to typify Tanzania or Mohc in any category because each of the three plots fell into a different quadrant (Fig. 6). Only two plots, corresponding to different cultivars fell into the Sensitive category in GI treatment, both close to the borderline to other categories. Under NI treatment, 58.3, 25.0, 13.9, and 2.8 % of the plots fitted to Sensitive, Resistant, Susceptible, and Tolerant categories, respectively (Table S4).

**Fig. 7:**
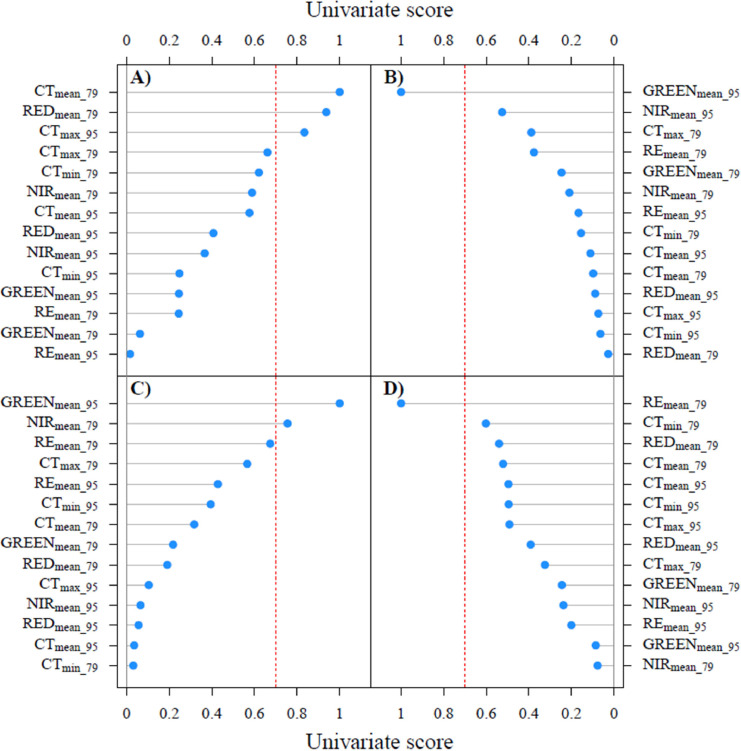
Relative importance of the effect of 14 explanatory predictors using SVM algorithm on sweetpotato virus resistance classification: A) Susceptible, B) Tolerant, C) Sensitive, and D) Resistant. GREEN, NIR, RE, and CT correspond to reflected spectral bands green (560 ± 20 nm), near-infrared (840 ± 40 nm), red-edge (717 ± 10 nm), and apparent canopy temperature, respectively. Subscripts “mean”, “min”, and “max” indicate the average, minimum, and maximum values of all pixels contained in each plot, respectively. Subscripts 79 and 95 indicate that the assessment was made at 79 and 95 days after transplanting, respectively. Red dashed line means a univariate score of 0.7.

Support vector machine showed the highest Argmax value of the global system accuracy for the testing stage, closely followed by random forest and K-nearest neighbors (Table 1). The accuracy per category in the testing stage ranged between 0.58 - 0.89 for the three algorithms used, and the minimum and maximum values were detected in the Resistant and Susceptible categories, respectively (Table 1). These outcomes represent the mean values obtained from 20 iterations, each conducted with distinct datasets randomly chosen from a 33 % subset of the total data (testing data). In the training stage (data not shown), random forest returned accuracies of 1 for all the four categories, which was interpreted as overfitting, unlike support vector machine and K-nearest neighbors. In addition, support vector machine gave close values of overall accuracy by category in the training and testing procedures. The best predictors (feature univariate score > 0.70) to discriminate Susceptible category were the mean (assessed at 79 DAT) and maximum (assessed at 95 DAT) value of canopy temperature, as well as mean red band assessed at 79 DAT (Fig. 7A). For the Tolerant and Sensitive categories, the best predictor was mean green band assessed at 95 DAT, and additionally mean near-infrared band assessed at 79 DAT for Sensitive category only (Figs. 7 B, C). Mean red-edge band assessed at 79 DAT showed as the best predictors for the Resistant category (Fig. 7D).

**Table 1:** Accuracy per category and Argmax value for the global system accuracy (Overall) on sweetpotato genotypes classification using random forest (RF), support vector machine (SVM) and k-nearest neighbors (KNN) algorithms.

Algorithm	Resistant	Susceptible	Tolerant	Sensitive	Overall
RF	0.81	0.89	0.88	0.73	0.84
SVM	0.58	0.78	0.89	0.75	0.87
KNN	0.71	0.89	0.88	0.73	0.82

## 4 Discussions

### 4.1 Classical vs. quantitative virus susceptibility classification in sweetpotato

Currently, breeders measure virus susceptibility as a qualitative trait in a range from 1 to 9 in field evaluations based on virus symptoms from the early to the last stages of the breeding process (Grüneberg et al., 2019). In this categorization, a good yielding (resistant) clone is defined by a threshold (set by control clones) and not by the comparison with a non-infected clone. Thus, the current method is unable to distinguish between Resistant and Tolerant, or Susceptible and Sensitive genotypes as defined in this study, even if Resistance and Tolerance components undoubtedly contribute to the resistance breeders observe in the field. To the best of our knowledge, it has not previously been systematically investigated across an assortment of genotypes ranging in susceptibility/resistance, to what extent viral symptoms correlate with yields or virus loads in plants. In our experiment, by design we included such a range of genotypes according to breeders’ characterizations based on symptoms and yield (Table S1). When considering relative viral loads as determined by (RT-)qPCR from pre-infected plants, the breeders’ categories were only partially consistent as reflected in virus titres and symptoms scores (Fig. 4). Notably, cultivar ‘Arne’ and ‘Dagga’ considered as resistant and moderately resistant by breeders, behaved as Susceptible when subjected to a combination of three viruses, with a maximum symptom score of 9 and average RER scores for SPFMV and SPCSV >10^3^ above the calibrator. This suggests other mechanisms, such as resistance to infection by vectors, also contributing to the field resistance observed by breeders. This type of resistance will manifest itself only after several successive seasons under natural exposed conditions in the field, as fewer plants become infected and manifest symptoms over time and could not be captured in our trial that lasted only one season. On the other hand, genotype VZ08.391 and ‘Ejumula’, considered resistant and susceptible by breeders, respectively, fell into the Tolerant category (Fig. 6) in our experiment when graft infected. Ejumula is a popular landrace in East Africa where virus pressure is prevalent and extremely high when breeders have assessed this variety (Mwanga et al., 2007). Ejumula was not among the high yielding varieties under San Ramon conditions, even when uninfected, so even if it's yield reduction might not be much after infection, breeders would have considered it susceptible if it yielded less than their check varieties, even if it is clearly not as susceptible as varieties grown in areas with low virus pressure such as ‘Beauregard’ (McGregor et al, 2009) and ‘Benjamin’. Genotype VZ08.328, considered moderately resistant by breeders (David et al., 2023) manifested as Tolerant in our experiment (Figs. 4 and 6). It also represents a rare genotype that seems to be susceptible to SPCSV but resistant to the synergistic increase of SPFMV and thus SPVD, which has not previously been reported and could have the potential to be exploited as a resistance mechanism. Further study of this genotype to determine the mechanism of synergism avoidance, which could be speculated to result from lack of interaction of RNase3 with SGS3 (Weinheimer et al., 2016) or yet unidentified other sweetpotato proteins, could be merited to identify resistant alleles. Similarly, VZ08.294 displayed a unique level of resistance to SPLCV that could be exploited. In tomato, several begomovirus resistance genes have been identified, three of which correspond to RNA dependent RNA polymerases involved in transcriptional gene silencing of DNA, which is the main silencing mechanism targeting DNA viruses, whereas one corresponds to an mRNA surveillance factor and one to a classical nucleotide binding site leucine rich repeat (NBS-LRR) resistance gene (Voorburg et al., 2020). New Kawogo and SPK004 are considered the most SPVD resistant genotypes available (Gasura & Mukasa, 2010; Mwanga et al., 2002b; Mwanga et al., 2007) and this was confirmed in this experiment based on symptoms, viral loads and total yield loss (Figs. 4 and 6), particularly SPK004 had extremely low virus loads for SPFMV and SPCSV, but also SPLCV. Whereas New Kawogo has been previously analyzed genetically for SPVD resistance identifying two separate recessive loci (but the candidate genes have not been identified; Mwanga et al., 2002a), SPK004 has not been analyzed in the same way. The current availability of sweetpotato genome assemblies and sequencing based genotyping & expression profiling methods provide more powerful tools to identify the sweetpotato genes and alleles involved in the resistances identified in this study. Nevertheless, the complexity of the highly heterozygous hexaploidy sweetpotato genome makes identifying genes and alleles involved in resistance exceptionally challenging.

We were unable to identify clearly sensitive genotypes (characterized by low virus load but high symptoms or yield loss) in this study, suggesting that this is not a common phenotype in sweetpotato. Regardless, the results of this study have been able to dissect in more granularity the mechanisms of resistance that are being selected for by breeders, providing options to exploit them individually more effectively in the future.

### 4.2 Remote sensed imagery and Artificial Intelligence allowed to classify sweetpotato response to virus attack

Leaf reduction and deformation, vein clearing or mosaic and stunting of plants are general symptoms of virus attack in sweetpotato (Moyer & Salazar, 1989). SPCSV by itself can cause symptoms including yellowing and purpling of older leaves and stunting of the plants, symptoms which can often be confused with nutritional deficiencies (Gibson & Aritua, 2002). Infection by SPLCV and SPFMV by themselves are largely symptomless in sweetpotatoes (Gibson et al., 1997; Wanjala et al., 2020). Both viruses are synergized by SPCSV (Karjeija et al., 2000a; Cuellar et al., 2015) and in the case of SPFMV, often leads to severe symptoms and yield loss. The mean value of the red band (650-680 nm) and canopy temperature were the most prominent attributes for the Susceptible categories (Fig. 7). The red band is associated with chlorophyll content and damage to the photosynthetic apparatus caused by virus infection (Shi et al. 2019). Consistent with this, sweetpotato plants affected by SPVD exhibit reduced chlorophyll content and photosynthetic capacity (Njeru et al., 2004; Wang et al., 2019a) and a downregulation in genes related to photosynthetic pathways (Bednarek et al., 2021; Zhang et al., 2020) likely contributing to lower overall yield. Red band is included as a key wavelength to differentiate levels of viral infection in tobacco (Krezhova et al., 2012). In the case of canopy temperature, plant foliage with severe virus symptoms maintains a high temperature caused by stomatal closure (Wang et al., 2019a). High stomata closure is associated to a low ^13^C isotope discrimination in plants (Farquhar et al., 1989), which was in accordance with the negative relationship found between canopy temperature and Δ under the GI virus infection condition (Fig. 3C). The study´s findings suggested that sweetpotato genotypes strongly affected by virus infection rise their canopy temperature, likely caused by stomatal closure. Tolerant genotypes can reduce the effect of pathogen attack, showing some mechanisms to endure the impact of the pathogen concentration (Pagán & García-Arenal, 2020). Leaf greenness duration and extension can promote a higher photosynthetic activity providing an indirect tolerance mechanism associated to virus tolerance in plants (Paudel & Sanfaçon, 2018). The green wavelengths strongly predicted the Tolerant and Sensitive categories related to plant vigor, canopy cover, and biomass density (Nguyen et al., 2021). The findings suggested that Tolerant sweetpotatoes could accumulate green leaves faster than others increasing their capability of C assimilation contrary to Sensitive individuals, which showed lower plant vigor. The sensitive category was also predicted by near-infrared length wave (band absorbance from 820 nm up to 1025 nm) close related to leaf water status (Fahlgren et al., 2015) and considered critical for determining virus infection in other crops (Sinha et al., 2019; Wang et al., 2019b). Thus, Jinendra et al. (2010) found that the NIR spectrum was markedly sensitive to the latent soybean mosaic virus disease response 2 weeks before the appearance of visual symptoms on leaves. Previously, Owen (1958) presented that infection with tobacco mosaic virus decreased the water content in tobacco leaves. Because leaf expansion and growth require turgor pressure provided by leaf water content (Ali et al., 2023), the near-infrared signal in Sensitive sweetpotatoes could be related to reduced leaf growth. The major attribute associated to the Resistant category was the mean value of the red-edge band. Several studies have shown the relationship between indices calculated from the reflected RE band and chlorophyll content (Loayza et al., 2022; Sims & Gamon, 2002). Likewise, the chlorophyll content and reduction in leaf size were reported as indicators of resistant and susceptible lines to tomato leaf curl virus (Singh et al., 2019) and bean yellow mosaic virus (Arora et al. 2009). Previously, Marco (1975) stated that the determination of the relative chlorophyll content of tomato leaf curl virus infected plants can be used as objective method for selecting resistant lines in breeding programs. Resistance, or susceptibility to viruses in sweetpotato is generally thought to be the result of the efficiency of RNA silencing in different genotypes and the viruses abilities to counteract this defense (Cuellar et al., 2009, Kenesi et al., 2021, Rodamilans et al., 2018; Weinheimer et al., 2016). Such molecular processes that take place inside the cells may lead to disruption in normal cell functioning, leading to various symptoms (Wang et al., 2012), yet to our knowledge there are no studies published linking any of these to remotely sensible data that could further explain the importance of the different spectra observed in this study.

The temporal dynamics of sweetpotato canopy cover is characterized by fast initial leaf growth, followed by a point where maximum canopy cover is reached and remains stable under optimum conditions until harvest (Ramírez et al., 2017). This asymptotic behavior of canopy cover is disrupted when sweetpotato is submitted to non-optimum conditions, like drought stress, where the canopy is reduced because of senescence (Laurie et al., 2015; Lewthwaite and Triggs, 2012). Good canopy development is a prerequisite to enable appropriate signal collection using cameras attached to unmanned aerial vehicles (Barbedo, 2019; Ollinger, 2011; Xie & Yang, 2020). Therefore, Abdelbaki et al. (2021) highlight that the accuracy of retrieved data from unmanned aerial vehicles found better results when soil background is fully covered by crop cover even during partial/full cloud cover conditions. In a representative group of diverse sweetpotatoes from CIP gene bank, Ramírez et al. (2017) found that maximum canopy cover is achieved around 75-85 DAT. Assessing a panel of 24 sweetpotato genotypes under different water stress treatments in Mozambique, Ramírez et al. (2021) acquired canopy reflectance and thermal imagery using a unmanned aerial vehicles at 87 DAT at the time of maximum canopy cover. This period was close to the first time point (73 DAT) used by Heider et al. (2020) to assess heat stress response in a sweetpotato panel (1973 genotypes) using a drone. Most predictors with the largest influence on the classification used in this study were measured at 79 DAT (Fig. 7), a time close to the beginning of maximum canopy cover, highlighting the importance of phenotyping sweetpotato when this phenological stage is reached.

Similar studies evaluated the effectiveness of utilizing multispectral data obtained from unmanned aerial vehicles and machine learning algorithms for categorizing the distinct responses of 25 maize varieties to maize streak virus and centered on prediction of yields after maize streak virus infection rather than resistance to the virus *per-se* (Chivasa et al., 2020, 2021). In contrast to these works which focused solely on a single treatment involving plants inoculated with viruses, our approach encompasses two additional treatments: control and natural infection and utilized qPCR methods for the quantification of the virus load, complementing visual inspections. This enabled us to disaggregate overall genotype yield potential and virus resistance effects, including different categories of resistance. In terms of remote sensing data, we incorporate canopy temperature information, a parameter directly associated with Non-Photochemical Quenching (Müller et al., 2001) and stomatal conductance (Jones, 1999). Our machine learning algorithms exclusively utilized multispectral and thermal bands as features, deliberately omitting vegetation indices to prevent redundancy in the information. Additionally, while our dataset for training and validation was relatively small (108 samples), it exceeded the sample sizes found in previous works (Chivasa et al., 2020, 2021). Thus, we expect our approach will result in a more useful tool for selection of resistant genotypes in a breeding program.

### 4.3 LAMP facilitated a rapid virus load assessment with proper accuracy for two viruses

ELISA is mostly used for detection and/or quantification of sweetpotato viruses due to its robustness, low cost, and ease of use (Karjeija et al., 2000b; David et al., 2023). However, due to the presence of inhibitors and often low virus concentrations in sweetpotato, ELISA lacks the sensitivity needed to detect and quantify viruses for practical use and needs complementary assays for confirmation, most commonly involving grafting onto indicator plants and/or PCR (Moyer & Salazar, 1989). This is time consuming and does not provide quantitative results, therefore it is not considered appropriate for use in high throughput resistance screening. Besides, antibodies are not available for all major viruses (like SPLCV). (RT-)qPCR is widely accepted as the gold standard for highly sensitive and quantitative determination of pathogen loads and several assays have also been developed for the most important sweetpotato viruses (Kokkinos & Clark, 2006; Varela-Benavides & Trejos-Araya, 2020). However, (RT-)qPCR is expensive and requires a relatively sophisticated laboratory for RNA extraction. LAMP is an alternative nucleic acid amplification method that is nearly as sensitive as qPCR, but more straightforward to use because the enzyme is tolerant to inhibitors and crude sample preparations can be used avoiding the need of nucleic acid extractions. LAMP assays have been developed and validated for the three sweetpotato viruses used in this study (Wanjala et al., 2021). To evaluate if LAMP could be used as a cheaper, routine substitute for qPCR for the quantitative determination of virus load in genotypes to support virus resistance screening, we analyzed the same sample in the same equipment by both methods using a simple measure corresponding to the inverse C(t) to estimate relative virus loads in the sample. When the exact same kit extracted RNA was used a clear and high correlation of 0.89 and 0.88 between LAMP and qPCR inverse C(t)’s was observed for SPFMV and SPCSV, respectively (Figs. 5A, B), indicating that LAMP could function as a useful alternative to qPCR for these two viruses that is more amenable to high throughput use. This direct comparison was done using kit extracted RNA due to limitations presented in our field experiment, but from the separate experiment with leaves ground into a homogenized powder we show that LAMP performance is effectively identical from kit extracts or alkaline polyethylene glycol extract (Fig. S3), signifying that the same high correlation should be expected if alkaline polyethylene glycol is used. The low correlation in the case of SPLCV requires further research to understand, but it could be related to differences in target regions of the SPLCV genome of the two assays, the qPCR targeting the intergenic region while LAMP primers target C1 encoding region, and possible interferences from defective DNAs which can be abundant in SPLCV infected plants (Paprotka et al., 2010).

## 5 Conclusions

The combination of virus load and yield reduction assessments provided a quantitative framework to classify sweetpotato genotypes into four categories according to their response to virus infection, discriminating Resistance from Tolerance, and Susceptible from Sensitive. Although this may represent an oversimplification of reality where genotypes will vary on a continuous scale between these categories, such categorization was useful and effective to enable training of machine learning algorithms to classify genotypes. This study's findings clearly demonstrated the utility of artificial intelligence algorithms applied to multispectral reflectance and thermal emissions acquired by sensors attached to unmanned aerial vehicles to facilitate phenotyping of virus resistance on a large panel of genotypes for breeding purposes. Where it is not possible to use unmanned aerial vehicles, such sensors can be attached to scaffolds above the plant canopy. This provides a possibility to implement a high throughput phenotyping approach based on quantitative data to select for virus resistance in sweetpotato. Phenotyping using remotely sensed data could be further complemented by using fast and sensitive LAMP assays to confirm results from remotely sensed classifications, as we demonstrated in the research that C(t) values of LAMP and qPCR have a reasonable correlation to distinguish plants with high and low viral loads. Current breeding approaches are based on symptoms and yield compared to check clones rather than compared to uninfected controls, whereas this study has shown that remotely sensed data can identify these categories and should be applicable in breeders’ selection plots where they rely on natural infection over several seasons. We anticipate that the approach described would lead to higher genetic gains and to the accelerated development of virus resistant sweetpotato varieties serving the needs of smallholder farmers in Africa and other geographies with a strong pressure of sweetpotato viruses.

## CRediT authorship contribution statement

**Jan F. Kreuze:** Conceptualization, Project administration, Supervision, Formal analysis, Investigation, Methodology, Writing – original draft, Writing – review & editing. **David A. Ramírez:** Conceptualization, Project administration, Supervision, Formal analysis, Methodology, Writing – original draft, Writing – review & editing. **Segundo Fuentes:** Project administration, Supervision, Resources, Data curation, Formal analysis, Investigation, Methodology, Writing – original draft, Writing – review & editing. **Hildo Loayza:** Supervision, Data curation, Software, Formal analysis, Investigation, Methodology, Writing – original draft, Writing – review & editing. **Johan Ninanya:** Data curation, Formal analysis, Visualization, Writing – review & editing. **Javier Rinza:** Data curation, Formal analysis, Investigation, Writing – original draft, Writing – review & editing. **Maria David:** Resources, Investigation, Writing – original draft, Writing – review & editing. **Soledad Gamboa:** Investigation. **Bert De Boeck:** Visualization, Formal analysis. **Federico Diaz:** Resources, Investigation, Writing – review & editing. **Ana Pérez:** Investigation. **Luis Silva:** Investigation. **Hugo Campos:** Funding acquisition, Writing – review & editing.

## Declaration of Competing Interest

The authors declare that they have no known competing financial interests or personal relationships that could have appeared to influence the work reported in this paper.

## Data Availability

All data has been published in dataverse, where it can be freely accessed: https://doi.org/10.21223/ECUF6I, https://doi.org/10.21223/269Z3N.

## Appendix Supplementary materials

Image, application 1
