# Supplementary material for: High-throughput characterization and phenotyping of resistance and tolerance to virus infection in sweetpotato
Source: Virus Res. 2023 Nov 25;339:199276. doi: 10.1016/j.virusres.2023.199276 (PMC10751700; doi:10.1016/j.virusres.2023.199276)
Supplement: Supplementary file 1 [file mmc1.docx]

**Table S1.** Selected accessions from International Potato Center genebank and improved varieties used in this study..

| **Accession Number** | **Accession Name** | **DOI** | **Country of Origin** | **Breeders assessed virus reaction** | **Biological status** |
| --- | --- | --- | --- | --- | --- |
| [CIP 440166](https://genebank.cipotato.org/gringlobal/accessiondetail.aspx?id=9255) | Tanzania | 10.18730/66NY | Uganda | Moderate | Traditional cultivar/landrace |
| [CIP 441745](https://genebank.cipotato.org/gringlobal/accessiondetail.aspx?id=7196) | New Kawogo | 10.18730/7ED7 | Uganda | Resistant | Traditional cultivar/landrace |
| [CIP 440132](https://genebank.cipotato.org/gringlobal/accessiondetail.aspx?id=9209) | Beauregard | 10.18730/65R1 | USA | Susceptible | Variety |
| [CIP 443750](https://genebank.cipotato.org/gringlobal/accessiondetail.aspx?id=6744) | Ejumula | 10.18730/V1C71 | Uganda | Susceptible | Traditional cultivar/landrace |
| [CIP 440034](https://genebank.cipotato.org/gringlobal/accessiondetail.aspx?id=9111) | Mohc | 10.18730/P670X | Burundi | Moderate | Traditional cultivar/landrace |
| [CIP 443881](https://genebank.cipotato.org/gringlobal/accessiondetail.aspx?id=6875) | SPK004/1; Kakamega | 10.18730/7NPJ | Uganda | Resistant | Traditional cultivar/landrace |
| [CIP 105085.2](https://genebank.cipotato.org/gringlobal/accessiondetail.aspx?id=5375) | INIA 307; Benjamin | 10.18730/P79AH | Peru | Susceptible | Variety |
| [CIP 105086.1](https://genebank.cipotato.org/gringlobal/accessiondetail.aspx?id=5376) | INIA 308; Arne | 10.18730/P79BJ | Peru | Resistant | Variety |
| CIP107578.6 | VZ08.294 | NA | Peru | Susceptible | Advanced clone |
| CIP107586.8 | VZ08.391 | NA | Peru | Resistant | Advanced clone |
| CIP107581.17 | VZ08.328 | NA | Peru | Moderate | Advanced clone |
| CIP199062.1 | Dagga | NA | Peru | Moderate | Variety |

**Table S2** List of primers and probes used for detecting viruses by LAMP and qPCR.

| **Essay** | **Virus** | **Primers** | **Sequence (5’ – 3’)** |
| --- | --- | --- | --- |
|  | SPCSV | SPCSV_F3_A | CCGATTATGATGGTTCCGATT |
|  |  | SPCSV_B3_A | CGGCGAAAGTCTTCCTAC |
|  |  | H SPCSV_FIP_A | TGACATACGATGCGACAGCCGGAAGTCGTCATAGATTGGATT |
|  |  | H SPCSV_BIP_A | CGCGTATGCTGACAGATCTCTTATTATGAGCGCGAAGCAA |
|  |  | SPCSV_LF_A | CACCTGAAGTACAAATGCTGTG |
|  |  | SPCSV_LB_A | ATGCTGATGCTGAATCTCTGT |
|  | SPFMV | SPFMV_F3 | TACAACGTAAMCTTGACTGATATGAGT |
|  |  | SPFMV_B3 | GTTATGTATATTTCTAGTAACRTCAGT |
|  |  | H SPFMV_FIPv2 | TGCRGCTGCYTTCATCTGYAWWTGTGGATATGCATTTGATTTYTAYGAGCT |
| LAMP |  | H SPFMV_BIP | AAGAATGCGMRWAATCGGTTGTTTGGGCCTCTCCGTATCYTCTTCTT |
|  |  | SPFMV_LF | TTCTTTAGCACGTGYAGGKG |
|  |  | SPFMV_LB | TGGAYGGAAACGTCTCCAC |
|  | SPLCV | sweepo_F3 | TTGCCAGTCCTTCTGGGC |
|  |  | sweepo_B3 | GTAATTTAGATAGGATWTTTTCWCC |
|  |  | H sweepo_FIP | GAAGGCCCAAGYAGAATAGGCAATTTAGGTATTGGGGGTTGACGT |
|  |  | H sweepo_BIP | ATCCATSACATTYTCAGCRGCCCTCCTTCTGTITATTCTTCICCTT |
|  |  | sweepo_FL | TACAGCAACAGTGCTTGGTAT |
|  |  | sweepo_BL | ARTCRCTGATAATGTCAGGWAC |
|  | COX | COX F3 | TATGGGAGCCGTTTTTGC |
|  |  | COX B3 | AACTGCTAAGRGCATTCC |
|  |  | H COX FIP | ATGGATTTGRCCTAAAGTTTCAGGGCAGGATTTCACTATTGGGT |
|  |  | H COX BIP | TGCATTTCTTAGGGCTTTCGGATCCRGCGTAAGCATCTG |
|  |  | COX LF | ATGTCCGACCAAAGATTTTACC |
|  |  | COX LB | GTATGCCACGTCGCATTCC |
|  | SPCSV | SPCSV-Uni-E-P (Taqman Probe) | [FAM]-TCTGTCACGGCTACAGGCGACGTG-[TAMRA] |
|  |  | SPCSV-Uni-E-F | CGGAGTTTATTCCCACYTGTYT |
|  |  | SPCSV-Uni-E-R | GGGCAGCCYCACCAA |
|  | SPFMV | SPFMVuni 847P (Taqman Probe) | [6-FAM]-AACGTCTCCACGCAAGAAGAGGATGC-[TAMRA] |
|  |  | SPFMV-uni-818F | CGCATAATCGGTTGTTTGGTTT |
|  |  | SPFMV-uni-925R | TTCCTAAGAGGTTATGTATATTTCTAGTAACATCAG |
| qPCR | SPLCV | SPLCV-P (Taqman Probe) | [6-FAM]-TGTGGGACCCTTTGC-[MGB] |
|  |  | SPLCV-F | GGCGCCTAAGTATGGCTGAA |
|  |  | SPLCV-R | AACCGTATAAAGTATCTGGGAGTGTGT |
|  | COX | COX-P (Taqman Probe) | [VIC]-TGCTTACGCTGGATGGAATGCCCT-[TAMRA] |
|  |  | COX-F | CGTCGCATTCCAGATTATCCA |
|  |  | COX-R | CAACTACGGATATATAAGAGCCAAAACTG |

**Table S3**. One-tube step of master mix reaction for qPCR and LAMP tests.

| **Essay** | **Reagents^a^** | **Quantity per**  **reaction (**µ**l)** | **Final**  **concentration** |
| --- | --- | --- | --- |
| qPCR | TaqMan Universal PCR Master mix (2X) | 12.5 | 1X |
|  | Nuclease-free water (NFW) | 7.5 | - |
|  | Forward primer (10 µM) | 0.75 | 300 nM |
|  | Reverse primer (10 µM) | 0.75 | 300nM |
|  | Probe (5 µM) | 0.5 | 100 nM |
|  | M-MLV RT^b^ diluted 1/100 (2U/µl) | 1.0 | 2U |
|  | Total volume | 23 |  |
|  |  |  |  |
| LAMP | Isothermal Master mix (ISO-001) | 15 | 1X |
|  | Nuclease-free water (NFW) | 4.7 | - |
|  | Primer F3 (10 µM) | 0.5 | 200 nM |
|  | Primer B3 (10 µM) | 0.5 | 200nM |
|  | Primer FIB (100 µM) | 0.5 | 2 µM |
|  | Primer BIP (100 µM) | 0.5 | 2 µM |
|  | Primer FL (100 µM) | 0.25 | 1 µM |
|  | Primer BL (100 µM) | 0.25 | 1 µM |
|  | SuperScript III RT^c^ diluted 1/50 (4U/µl) | 0.8 | 3.2U |
|  | Total volume | 23 |  |

^a^ 2 µl of target was included in each reaction for reaching a final volume of 25 µl

^b^ Moloney Murine Leukemia Virus (M-MLV) reverse transcriptase-200U/µl (Invitrogen, USA)

^c^ SuperScript III reverse transcriptase-200U/µl (Invitrogen, USA)

**Table S4.** Total viral load values (TVL), percental variation of total yield to control (Δ%) and categories in each plot for the natural infected treatment (NI).

| **Genotype** | **Repetitions** | **TVL** | **Δ%** | **NI** |
| --- | --- | --- | --- | --- |
| Arne | 1 | 87384.8 | -52.8 | Susceptible |
| Arne | 2 | 753.6 | -37.0 | Sensitive |
| Arne | 3 | 5748.3 | -19.9 | Resistant |
| Beauregard | 1 | 255959 | -37.4 | Susceptible |
| Beauregard | 2 | 3837734.3 | -39.2 | Susceptible |
| Beauregard | 3 | 18.9 | -4.0 | Resistant |
| Benjamin | 1 | 444419.6 | -2.7 | Tolerant |
| Benjamin | 2 | 2085212.3 | -22.7 | Susceptible |
| Benjamin | 3 | 2250.1 | -34.5 | Sensitive |
| Dagga | 1 | 10481.7 | -30.4 | Sensitive |
| Dagga | 2 | 2032.4 | -44.3 | Sensitive |
| Dagga | 3 | 109113.7 | -28.3 | Susceptible |
| Ejumula | 1 | 14.2 | -35.5 | Sensitive |
| Ejumula | 2 | 49785.1 | -9.5 | Resistant |
| Ejumula | 3 | 42.8 | -34.2 | Sensitive |
| Mohc | 1 | 13.4 | -35.1 | Sensitive |
| Mohc | 2 | 803.7 | -17.8 | Resistant |
| Mohc | 3 | 3548.9 | -11.3 | Resistant |
| New Kawogo | 1 | 37.8 | -42.1 | Sensitive |
| New Kawogo | 2 | 54.5 | -37.3 | Sensitive |
| New Kawogo | 3 | 6.1 | -48.4 | Sensitive |
| SPK004 | 1 | 44.3 | -43.7 | Sensitive |
| SPK004 | 2 | 36.3 | -4.0 | Resistant |
| SPK004 | 3 | 6.9 | -32.4 | Sensitive |
| Tanzania | 1 | 6.9 | -4.5 | Resistant |
| Tanzania | 2 | 26.2 | -22.7 | Sensitive |
| Tanzania | 3 | 526.8 | -23.9 | Sensitive |
| VZ08.294 | 1 | 26158.5 | -43.2 | Sensitive |
| VZ08.294 | 2 | 14181.8 | -39.4 | Sensitive |
| VZ08.294 | 3 | 1947.8 | -30.4 | Sensitive |
| VZ08.328 | 1 | 38.5 | -22.8 | Sensitive |
| VZ08.328 | 2 | 202.9 | -26.5 | Sensitive |
| VZ08.328 | 3 | 52.5 | -17.5 | Resistant |
| VZ08.391 | 1 | 9998 | -18.8 | Resistant |
| VZ08.391 | 2 | 4645.7 | -33.7 | Sensitive |
| VZ08.391 | 3 | 18330.2 | -32.6 | Sensitive |


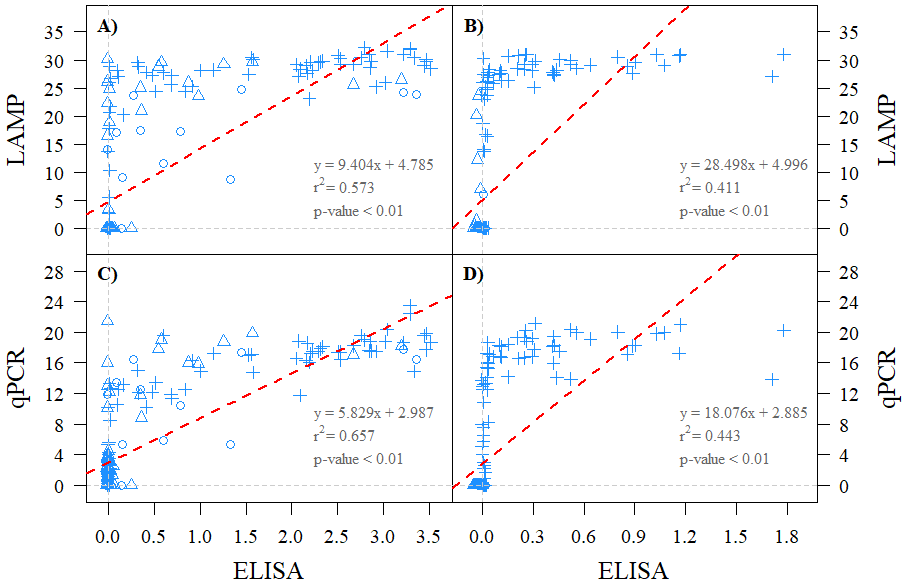


**Figure S1.** Correlation between inverse C(t) values of LAMP and qPCR methods with ELISA used for the detection of SPFMV (A and C) and SPCSV (B and D) in the graft-infected (+), natural infected (Δ), and control (○) treatments at 48 days after transplanting. Observe that large number of samples resulted negative to ELISA but positive to LAMP or qPCR.


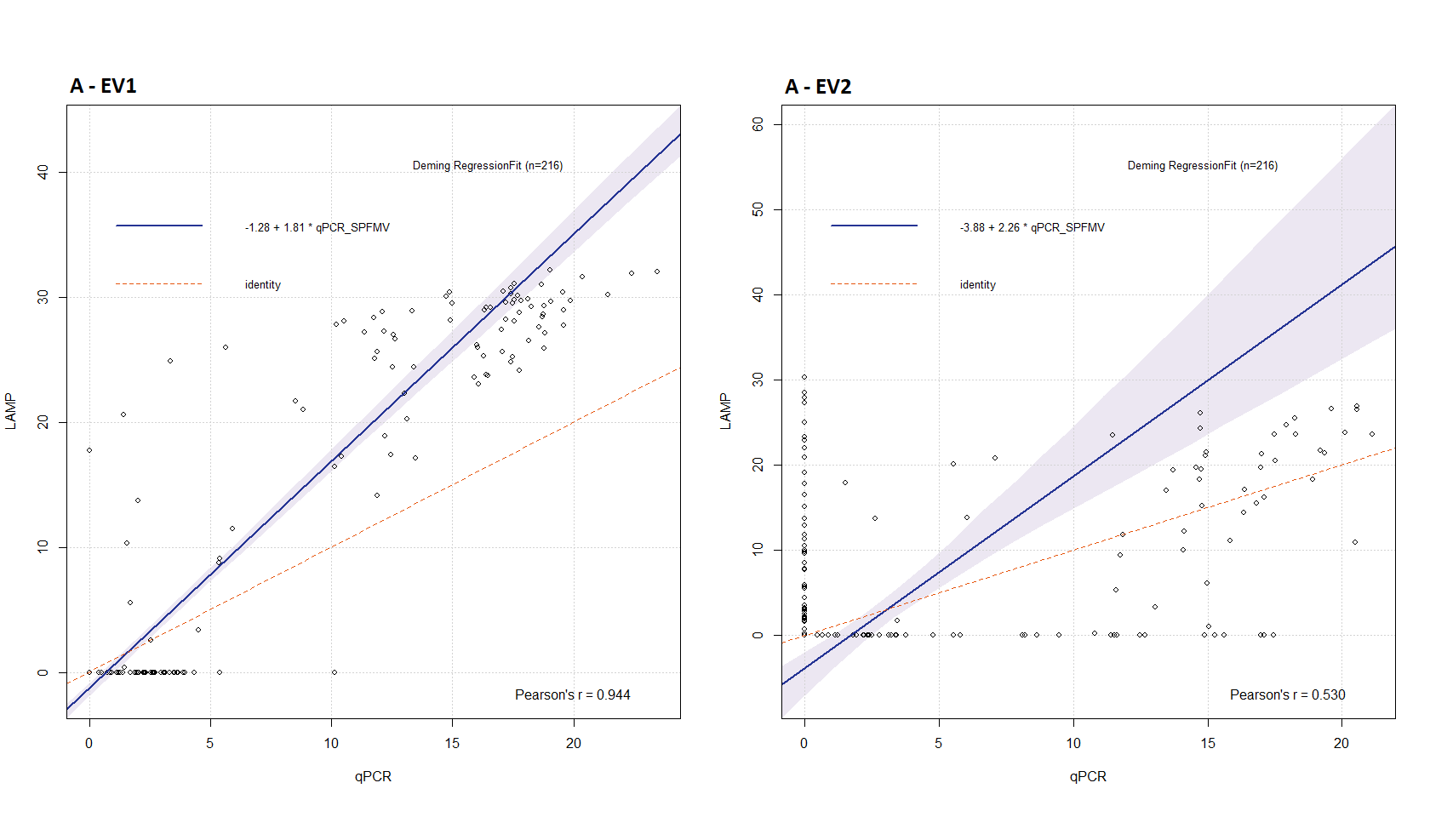


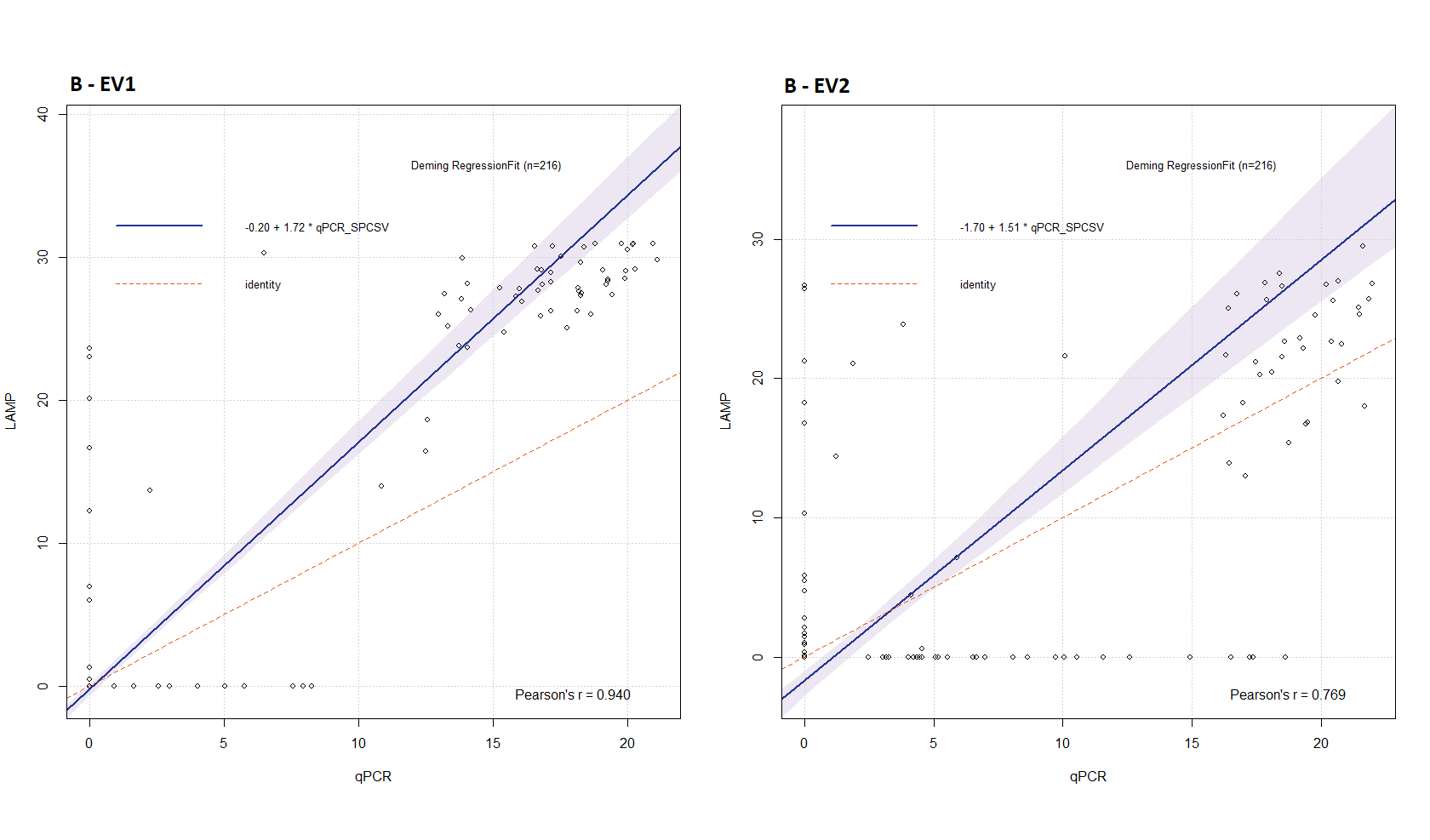


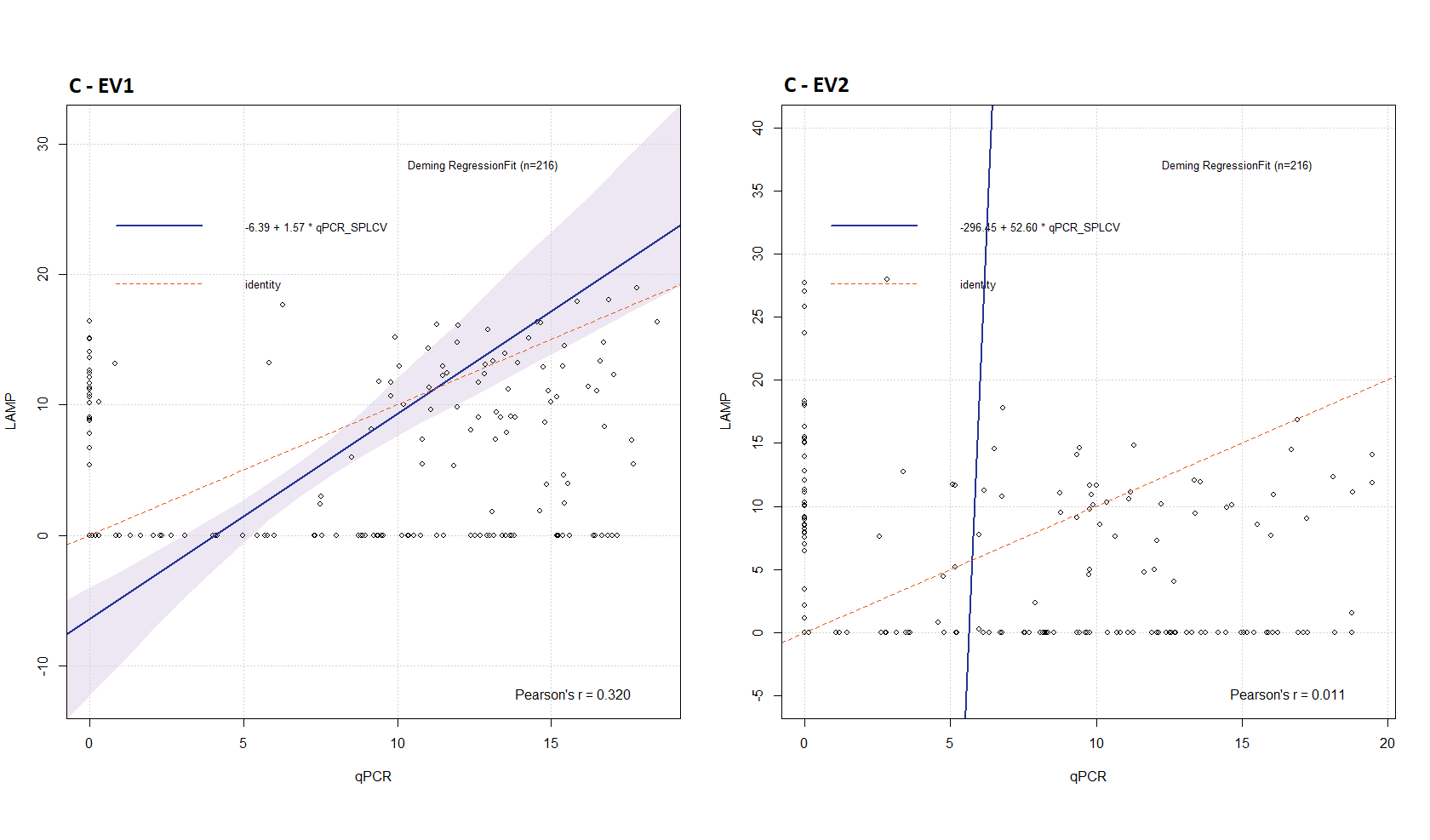


**Figure S2.** Fitted Deming regression lines describing LAMP versus qPCR (reference) measurement relationships for SPFMV (A), SPCSV (B) and SPLCV (C) viruses and both evaluation times (EV1 and EV2). The 95% confidence area for the regression line (blue) is represented in transparent blue (not shown for plot C-EV2 as the confidence area covers the entire chart).


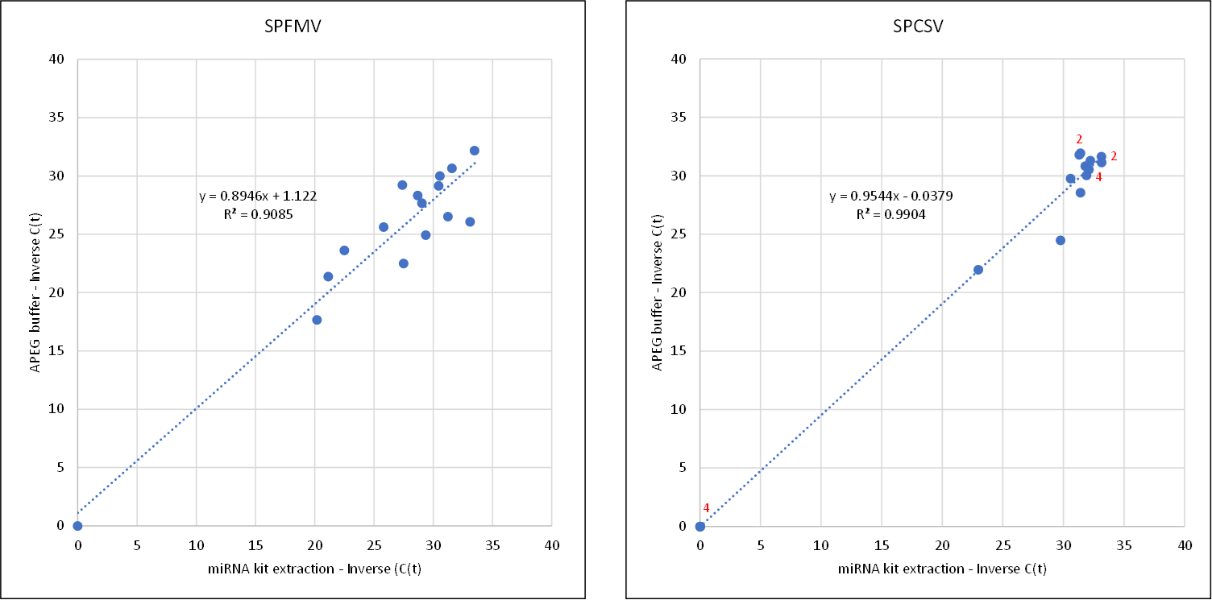


**Figure S3**. Correlation between inverse C(t) values of LAMP results performed for detecting SPFMV and SPCSV from identical samples extracted with APEG buffer and miRNA kit. Numbers in red color beside the blue spots in SPCSV graphic correspond to the number of spots in the same position or closely.
